# Generative AI as a Tool for Environmental Health Research Translation

**DOI:** 10.1029/2023GH000875

**Published:** 2023-07-26

**Authors:** Lauren B. Anderson, Dhiraj Kanneganti, Mary Bentley Houk, Rochelle H. Holm, Ted Smith

**Affiliations:** ^1^ Christina Lee Brown Envirome Institute School of Medicine University of Louisville Louisville KY USA; ^2^ Department of Urban and Public Affairs College of Arts and Sciences University of Louisville Louisville KY USA; ^3^ University of Louisville Superfund Research Center Louisville KY USA

**Keywords:** plain language summaries, artificial intelligence, environmental health justice

## Abstract

One valuable application for generative artificial intelligence (AI) is summarizing research studies for non‐academic readers. We submitted five articles to Chat Generative Pre‐trained Transformer (ChatGPT) for summarization, and asked the article's author to rate the summaries. Higher ratings were assigned to more insight‐oriented activities, such as the production of eighth‐grade reading level summaries, and summaries highlighting the most important findings and real‐world applications. The general summary request was rated lower. For the field of environmental health science, no‐cost AI technology such as ChatGPT holds the promise to improve research translation, but it must continue to be improved (or improve itself) from its current capability.

## Key Points

- Generative artificial intelligence (AI), popularized by services like Chat Generative Pre‐trained Transformer (ChatGPT), has been the source of much recent popular attention for publishing health research
- AI production of high‐quality plain language summaries could improve access to scientific information
- ChatGPT holds the promise to improve research translation, but it must continue to be improved from its current capability

## 1 Introduction

Generative artificial intelligence (AI), popularized by services like Chat Generative Pre‐trained Transformer (ChatGPT), has been the source of much recent popular attention for publishing health research (Liebrenz et al., 2023; van Dis et al., 2023). The software can instantly create sophisticated content ranging from text‐to‐image to text‐to‐audio and video depending on user prompts (Gozalo‐Brizuela & Garrido‐Merchan, 2023). ChatGPT, released by OpenAI (2023; San Francisco, CA, USA) in November of 2022, is a large language model (LLM) trained on a vast amount of data that generates human‐like responses to queries. One potentially valuable application is the summarization of studies in support of research translation intended for non‐specialist readers. The field of environmental health sciences exemplifies this opportunity given the specialized language surrounding environmental contamination which often relies on knowledge of chemistry, toxicology, epidemiology, and physical sciences. Very often, concerned members of the public are excluded from that primary literature due to these barriers. These might include environmental justice communities, mainstream media outlets, and community science groups. Plain language summaries (PLSs) for a nonexpert audience should improve on accessibility, understanding, knowledge, communication of research, and empowerment (Stoll et al., 2022). As a complement, research translation activities should include the identification of key messages and development of messages that are easily understood across a variety of audiences (Grimshaw et al., 2012). Given the number of challenges with writing peer‐reviewed research and the follow‐on cost and effort in additionally creating PLSs, generative AI may offer a valuable new tool for expanding dissemination of environmental health risk research and providing access to broader constituencies.

LLM generative AI such as ChatGPT could greatly advance environmental health research by making an important contribution to wider dissemination of PLSs to non‐experts who are often important in regulatory and other policy development. This should not be done uncritically but by vigilantly weighing the strengths and weaknesses of these applications of generative AI. On one hand, it could enhance fairness, diversity, and accessibility in science (van Dis et al., 2023). On the other hand, it may pose risks by undermining research quality and trust. Chatbots can produce convincing but inaccurate text, which might undermine the very trust that research summarization aims to build (Liebrenz et al., 2023; van Dis et al., 2023). The role of academic researchers in environmental health programs is not limited to methods, but also the essential working relationships across audiences for translation to public health action (Hoar et al., 2022). We explored the use of ChatGPT as an alternative to traditional human expert written summarization to support PLSs as a form of research translation and provide suggestions for environmental health research summarization.

## 2 Methods

A convenience sample of five peer‐reviewed environmental health articles were used to generate four different summaries using generative AI (OpenAI, 2023). The inclusion criteria for the selected manuscripts were: recently published (2021–2022), open‐access, peer‐reviewed published articles, and authored by colleagues who were University of Louisville environmental health investigators and collaborators with relevance to the general public (Supporting Information S1). The PLSs were created by entering the full text of each article into the ChatGPT interface with the prompt: “read the following,” followed by a series of prompts: “Summarize the findings of this study in 500 words,” “Summarize the research paper at an eighth‐grade reading level,” “What is the most important finding of this study?” and “What are the real‐world impacts of this study?.” These prompts were selected because they reflect the effort to identify each study's key messages for research translation purposes (Grimshaw et al., 2012), the intended audience of the ChatGPT‐generated summaries was a non‐expert. The level of an eighth grade readability was chosen as a metric of comprehension comparable to a typical community newspaper. Study authors who were either first, senior, or corresponding were contacted to review the summarizations. The author was provided rating instructions (Supporting Information S1) and rated each summary one time. The responses from ChatGPT were evaluated using a combination of Likert‐scale, yes/no, and text to assess scientific accuracy, completeness, and readability at an eighth grade level by one study author. The author reviewers were blinded to the nature of the summarization; instructions stated that “summaries were written about their work.” The use of generative AI was not disclosed nor was brought up to the authors conducting the ratings. Due to design limitations of using an author of each paper as rater, inter‐rater reliability could not be assessed.

## 3 Results and Discussion

The average rating of summaries across the five studies ranged between 3 and 5 (average rating of 3.9). A score of 1 indicated poor quality and where 5 indicated good overall content quality. ChatGPT's general summary request was consistently rated lower than the other summary types (average rating of 3.4). Higher ratings were assigned to the more synthetic, insight‐oriented activities, such as identifying the most important finding (average rating of 4.1), the production of a PLS suitable for an eighth grade reading level (average rating of 3.9), and real‐world research applications (average rating of 3.9). These more insight‐orientated summaries were also judged acceptable for use with the public. Even across this limited set of articles, two authors commented that language used by the ChatGPT‐generated PLS was still too technical for general audiences. For instance, ChatGPT‐generated content stated “controlled for the potential confounding effects of race and wealth” or that methods using “geographically clustered probability sampling design is representative of the US civilian non‐institutionalized population.” However, in some cases, ChatGPT's attempt at simplification removed important detail—for instance, stating wellbeing measures were associated with cardiovascular disease, rather than risk of cardiovascular disease. Minor inaccuracies around study method interpretations were also observed. In one article the ChatGPT‐generated PLS referenced questions about levels of pollution and greenness around the home when the study did not include these questions. This finding on inaccuracies is similar to medical discharge summaries created with automation which have been shown to still require manual checking by a human expert since an inaccuracy in a summary report may lead to patient safety issues (Patel & Lam, 2023). The current discourse in scientific writing presents concerns about the potential for AI to generate human‐like responses, thus bringing up the need for writing source disclosure (Gao et al., 2023).

There is a cost (economic, effort and time) of producing PLSs, for example, a Cochrane PLS is reported to take about 5 hr to write (Pitcher et al., 2022). In contrast, the no‐cost ChatGPT summary requests in this study took only seconds to generate. In an age of misinformation (Spitale et al., 2023), ChatGPT‐generated summaries may also disincentivize non‐experts to understand environmental health data. Errors such as disease association rather than risk‐for‐disease association could be quite troublesome.

In academic settings, while ChatGPT has been listed as a co‐author (Frye, 2023) it has also been argued that this should not apply because AI cannot be held accountable for scientific writing (van Dis et al., 2023) or contacted for further information to understand environmental health data. There also needs to be discussion around the notion of copyright and implications for Fair Use laws. The publishers own the copyright to these articles, and unless they are open access there is a potential gray area with employing ChatGPT (itself from a private company) to generate new content from this existing and copyrighted content even for research translation use.

## 4 Conclusion

There are several critical, yet under‐delivered, research needs, especially around inclusion and environmental health justice research (van Dis et al., 2023). This is a case where AI might do more good than harm and help level the playing field, for example, by creating accessible insights and enabling the large‐scale production of high‐quality PLSs which could improve open access to scientific information. This possibility, combined with the increasing public policy trends encouraging open access for research supported with public funds, may alter the role journal publications play in communicating science in society. Recommendations for future research include adding ratings by community members of paired (human vs. AI) PLSs; increasing the number and variety of environmental health research study types with more reviewers; setting safeguards to guard against the spread of misinformation; determining whether AI PLSs are culturally appropriate; and studying the efficiency of the AI PLS method. Having ratings done by an author of the article is suited to evaluating accuracy but may not be the best choice for other characteristics. The authors of this study did not consider the potential interpretability from a diverse set of audiences including communities that may be impacted by the research more than others. While not within the scope of this review, this would be an important caution and future direction to consider when analyzing the utility of ChatGPT for advancing translation of research for a broader lay audience. For the field of environmental health science, no‐cost AI technology such as ChatGPT holds the promise to improve research translation, but it must continue to be improved (or improve itself) from its current capability.

## Conflict of Interest

The authors declare no conflicts of interest relevant to this study.

## Supporting information

Supporting Information S1

## Data Availability

The list of peer‐reviewed articles analyzed during the current study are available in the supplement to this manuscript.
