# Supplementary material for: Generative AI as a Tool for Environmental Health Research Translation
Source: Geohealth. 2023 Jul 26;7(7):e2023GH000875. doi: 10.1029/2023GH000875 (PMC10369501; doi:10.1029/2023GH000875)
Supplement: Supplementary file 1 — Supporting Information S1 [file GH2-7-e2023GH000875-s001.docx]

**Supporting Information**

**Generative AI as a Tool for** **Environmental Health Research Translation**

Lauren B. Anderson,^1,2^ Dhiraj Kanneganti,^1^ Mary Bentley Houk,^1^ Rochelle H. Holm^1^ and Ted Smith^1,3*^

^1^Christina Lee Brown Envirome Institute, School of Medicine, University of Louisville, Louisville, KY 40202, United States

^2^Department of Urban and Public Affairs, College of Arts and Sciences, University of Louisville, Louisville KY 40208, United States

^3^University of Louisville Superfund Research Center, Louisville, KY 40202, United States

**Correspondence**

Correspondence should be sent to Ted Smith, Superfund Research Center, School of Medicine, University of Louisville, 302 E Muhammad Ali Blvd, Louisville, KY 40202, United States (e-mail: [ted.smith@louisville.edu](mailto:ted.smith@louisville.edu)).

Table of Contents

[List of peer-reviewed articles authored by University of Louisville Envirome Institute environmental health investigators and collaborating research partners entered into the ChatGPT interface: 3](#_Toc138738570)

[Table S1 Rubric for author evaluations 4](#_Toc138738571)

[Table S2. Evaluation responses from studied peer-reviewed articles 8](#_Toc138738572)

[References 13](#_Toc138738573)

# List of peer-reviewed articles authored by University of Louisville Envirome Institute environmental health investigators and collaborating research partners entered into the ChatGPT interface:

1. Coleman, C. J., Yeager, R. A., Riggs, D. W., Coleman, N. C., Garcia, G. R., Bhatnagar, A., & Pope, C. A. (2021). Greenness, air pollution, and mortality risk: A US cohort study of cancer patients and survivors. Environment International, 157, 106797. https://doi.org/10.1016/j.envint.2021.106797
2. Coleman, C. J., Yeager, R. A., Pond, Z. A., Riggs, D. W., Bhatnagar, A., & Pope III, C. A. (2022). Mortality risk associated with greenness, air pollution, and physical activity in a representative US cohort. Science of The Total Environment, 824, 153848. https://doi.org/10.1016/j.scitotenv.2022.153848
3. El-Mallakh, T. V., Hedges, S., Rai, J. P., Bhatnagar, A., Moyer, S., & El-Mallakh, R. S. (2022). Suicide and Homicide More Common with Limited Urban Tree Canopy Cover. Cities and the Environment (CATE), 14(2), 4. https://doi.org/10.15365/cate.2022.140204
4. McLeish, A. C., Smith, T., Riggs, D. W., Hart, J. L., Walker, K. L., Keith, R. J., ... & Bhatnagar, A. (2022). Community‐Based Evaluation of the Associations Between Well‐Being and Cardiovascular Disease Risk. Journal of the American Heart Association, 11(22), e027095. https://doi.org/10.1161/JAHA.122.027095
5. Pfeiffer, J. A., Hart, J. L., Wood, L. A., Bhatnagar, A., Keith, R. J., Yeager, R. A., ... & Walker, K. L. (2021). The importance of urban planning: Views of greenness and open space is reversely associated with self-reported views and depressive symptoms. Population medicine, 3. https://doi.org/10.18332/popmed/139173

# Table S1 Rubric for author evaluations

| **Author Evaluation Tool** | We are exploring how to make our research more accessible to the public. We have taken your paper, XXX, and summarized the findings in the following ways:  1. 500-word summary 2. Summary at an 8th grade reading level 3. The key takeaway 4. Real-world impacts |
| --- | --- |
| On a scale of 1-5, where 1 is low and 5 is high, please evaluate **the 500-word summary** for: | **Accuracy:** |
|  | 5 |
|  | 4 |
|  | 3 |
|  | 2 |
|  | 1 |
|  | **Comments:** |
|  |  |
|  | **Completeness:** |
|  | 5 |
|  | 4 |
|  | 3 |
|  | 2 |
|  | 1 |
|  | **Comments:** |
|  |  |
|  | **Readability:** |
|  | 5 |
|  | 4 |
|  | 3 |
|  | 2 |
|  | 1 |
|  | **Comments:** |
|  |  |
| **Would you consider this summary acceptable for use with the public?** | Yes / No |
|  |  |
|  | **Additional comments:** (Is there anything important missing from this summary? Any other feedback you’d like to add?) |
| On a scale of 1-5, where 1 is low and 5 is high, please evaluate **the summary at an 8th grade reading level** for: | **Accuracy:** |
|  | 5 |
|  | 4 |
|  | 3 |
|  | 2 |
|  | 1 |
|  |  |
|  | **Comments:** |
|  |  |
|  | **Completeness:** |
|  | 5 |
|  | 4 |
|  | 3 |
|  | 2 |
|  | 1 |
|  | **Comments:** |
|  |  |
|  | **Readability:** |
|  | 5 |
|  | 4 |
|  | 3 |
|  | 2 |
|  | 1 |
|  | **Comments:** |
|  |  |
| **Would you consider this summary acceptable for use with the public?** | Yes / No |
|  |  |
|  | **Additional comments:** (Is there anything important missing from this summary? Any other feedback you’d like to add?) |
| On a scale of 1-5, where 1 is low and 5 is high, please evaluate **the key takeaway statement** for: | **Accuracy:** |
|  | 5 |
|  | 4 |
|  | 3 |
|  | 2 |
|  | 1 |
|  | **Comments:** |
|  |  |
|  | **Completeness:** |
|  | 5 |
|  | 4 |
|  | 3 |
|  | 2 |
|  | 1 |
|  | **Comments:** |
|  |  |
|  | **Readability:** |
|  | 5 |
|  | 4 |
|  | 3 |
|  | 2 |
|  | 1 |
|  | **Comments:** |
|  |  |
| **Would you consider this statement acceptable for use with the public?** | Yes / No |
|  |  |
|  | **Additional comments:** (Is there anything important missing from this summary? Any other feedback you’d like to add?) |
|  |  |
|  |  |
| On a scale of 1-5, where 1 is low and 5 is high, please evaluate **the real-world impact statement** for: | **Accuracy:** |
|  | 5 |
|  | 4 |
|  | 3 |
|  | 2 |
|  | 1 |
|  | **Comments:** |
|  |  |
|  | **Completeness:** |
|  | 5 |
|  | 4 |
|  | 3 |
|  | 2 |
|  | 1 |
|  | **Comments:** |
|  |  |
|  | **Readability:** |
|  | 5 |
|  | 4 |
|  | 3 |
|  | 2 |
|  | 1 |
|  | **Comments:** |
|  |  |
| **Would you consider this statement acceptable for use with the public?** | Yes / No |
|  |  |
|  | **Additional comments:** (Is there anything important missing from this summary? Any other feedback you’d like to add?) |

# Table S2. Evaluation responses from studied peer-reviewed articles

|  | **Coleman et al., 2022** | **Coleman et al., 2021** | **El-Mallakh et al., 2022** | **McLeish et al., 2022** | **Pfeiffer et al., 2021** |
| --- | --- | --- | --- | --- | --- |
| 500 Word summary accuracy (1=poor quality, 5 good overall quality) | 3 | 1 | 4 | 4 | 3 |
| 500 Word summary completeness (1=poor quality, 5 good overall quality) | 2 | 3 | 4 | 5 | 4 |
| 500 Word summary readability (1=poor quality, 5 good overall quality) | 4 | 3 | 4 | 3 | 4 |
| 500 Word summary acceptable for public (1=Y, 0=N) | 1 | 0 | 1 | 1 | 0 |
| 500 Word summary comments | This summary is focusing on a lot of the methods without too much focus on the actual findings. And the limitations listed are not actually limitations of the study, but a summary of the null findings. | There are several major misinterpretations of the study. 1)The title of the paper is not correct. 2)The study is actually an individual level analysis, with each individual’s exposure levels estimated at the county level. 3)The study did not find a decrease in cardiopulmonary mortality associated with greenness in the full cohort of cancer patients, but they did find an association when stratifying to individuals with high survivability cancers. It also should have been noted that this is a study of cancer survivors, and the results do not necessarily apply to the general population. | It's repetitive | Potential of CVD and CVD risk. The language is still a little to technical/complex | Readability depends on audience (I assigned a 4 assuming an academic audience; if audience is general/community members, I'd shift that rating to a 3). |
| 8th Grade summary accuracy (1=poor quality, 5 good overall quality) | 3 | 4 | 5 | 3 | 3 |
| 8th Grade summary completeness (1=poor quality, 5 good overall quality) | 3 | 4 | 5 | 5 | 3 |
| 8th Grade summary readability (1=poor quality, 5 good overall quality) | 4 | 4 | 5 | 4 | 4 |
| 8th Grade summary acceptable for public (1=Y, 0=N) | 1 | 1 | 1 | 1 | 1 |
| 8th grade summary comments | There are some minor inaccuracies. The survey did not ask about the level of pollution or how green the area around their home was. |  |  | Was no finding on CVD but findings on CVD risk |  |
| Most important finding accuracy ((1=poor quality, 5 good overall quality) | 4 | 3 | 4 | 5 | 4 |
| Most important finding completeness (1=poor quality, 5 good overall quality) | 4 | 4 | 4 | 5 | 4 |
| Most important finding readability (1=poor quality, 5 good overall quality) | 5 | 5 | 4 | 3 | 4 |
| Most important finding acceptable for public (1=Y, 0=N) | 1 | 1 | 1 | 1 | 1 |
| Most important finding comments |  | The study found that greenness only protects against cardiopulmonary mortality in individuals with high survivable cancers, not for the full study. |  | A little too technical | Generally usable with public (but less so on last sentence, especially last phrase) |
| Real world impacts accuracy (1=poor quality, 5 good overall quality) | 4 | 4 | 4 | 2 | 5 |
| Real world impacts completeness (1=poor quality, 5 good overall quality) | 4 | 4 | 4 | 2 | 4 |
| Real world impacts readability (1=poor quality, 5 good overall quality) | 5 | 5 | 4 | 4 | 4 |
| Real world impacts acceptable for public (1=Y, 0=N) | 1 | 1 | 1 | 0 | 1 |
| Real word impacts comments |  |  | Would edit to mention importance of increasing tree canopy, and not just green spaces | They missed the application that was discussed on the paper |  |

# References

Coleman, C. J., Yeager, R. A., Riggs, D. W., Coleman, N. C., Garcia, G. R., Bhatnagar, A., & Pope, C. A. (2021). Greenness, air pollution, and mortality risk: A US cohort study of cancer patients and survivors. Environment International, 157, 106797. https://doi.org/10.1016/j.envint.2021.106797

Coleman, C. J., Yeager, R. A., Pond, Z. A., Riggs, D. W., Bhatnagar, A., & Pope III, C. A. (2022). Mortality risk associated with greenness, air pollution, and physical activity in a representative US cohort. Science of The Total Environment, 824, 153848. https://doi.org/10.1016/j.scitotenv.2022.153848

El-Mallakh, T. V., Hedges, S., Rai, J. P., Bhatnagar, A., Moyer, S., & El-Mallakh, R. S. (2022). Suicide and Homicide More Common with Limited Urban Tree Canopy Cover. Cities and the Environment (CATE), 14(2), 4. https://doi.org/10.15365/cate.2022.140204

McLeish, A. C., Smith, T., Riggs, D. W., Hart, J. L., Walker, K. L., Keith, R. J., ... & Bhatnagar, A. (2022). Community‐Based Evaluation of the Associations Between Well‐Being and Cardiovascular Disease Risk. Journal of the American Heart Association, 11(22), e027095. https://doi.org/10.1161/JAHA.122.027095

Pfeiffer, J. A., Hart, J. L., Wood, L. A., Bhatnagar, A., Keith, R. J., Yeager, R. A., ... & Walker, K. L. (2021). The importance of urban planning: Views of greenness and open space is reversely associated with self-reported views and depressive symptoms. Population medicine, 3. https://doi.org/10.18332/popmed/139173
